# Combining Microbubble Contrast Agent with Pulsed-Laser Irradiation for Transdermal Drug Delivery

**DOI:** 10.3390/pharmaceutics10040175

**Published:** 2018-10-03

**Authors:** Ai-Ho Liao, Ho-Chiao Chuang, Bo-Ya Chang, Wen-Chuan Kuo, Chih-Hung Wang, Hong-Wei Gao, Chien-Ping Chiang

**Affiliations:** 1 Graduate Institute of Biomedical Engineering, National Taiwan University of Science and Technology, Taipei 10607, Taiwan; aiho@mail.ntust.edu.tw; 2 Department of Biomedical Engineering, National Defense Medical Center, Taipei 11490, Taiwan; 3 Department of Mechanical Engineering, National Taipei University of Technology, Taipei 10608, Taiwan; hchuang@ntut.edu.tw (H.-C.C.); 40071103@gm.nfu.edu.tw (B.-Y.C.); 4 Institute of Biophotonics, National Yang-Ming University, Taipei 11221, Taiwan; wckuo@ym.edu.tw; 5 Biophotonics and Molecular Imaging Research Center, National Yang-Ming University, Taipei 11221, Taiwan; 6 Graduate Institute of Medical Sciences, National Defense Medical Center, Taipei 11490, Taiwan; chw@ms3.hinet.net; 7 Department of Otolaryngology-Head and Neck Surgery, Tri-Service General Hospital, National Defense Medical Center, Taipei 11490, Taiwan; 8 Taichung Armed Forces General Hospital, Taichung 41168, Taiwan; 9 Department of Pathology, Tri-Service General Hospital, National Defense Medical Center, Taipei 11490, Taiwan; doc31796@gmail.com; 10 Department of Dermatology, Tri-Service General Hospital, National Defense Medical Center, No. 325, Sec. 2, Chenggong Rd., Neihu District, Taipei 11490, Taiwan; 11 Department of Biochemistry, National Defense Medical Center, Taipei 11490, Taiwan

**Keywords:** ultrasound contrast agents, laser, transdermal, cavitation, arbutin

## Abstract

The optodynamic process of laser-induced microbubble (MB) cavitation in liquids is utilized in various medical applications. However, how incident laser radiation interacts with MBs as an ultrasound contrast agent is rarely estimated when the liquid already contains stable MBs. The present study investigated the efficacy of the laser-mediated cavitation of albumin-shelled MBs in enhancing transdermal drug delivery. Different types and conditions of laser-mediated inertial cavitation of MBs were first evaluated. A CO_2_ fractional pulsed laser was selected for combining with MBs in the in vitro and in vivo experiments. The in vitro skin penetration by β-arbutin after 2 h was 2 times greater in the group combining a laser with MBs than in the control group. In small-animal experiments, the whitening effect on the skin of C57BL/6J mice in the group combining a laser with MBs on the skin plus penetrating β-arbutin increased (significantly) by 48.0% at day 11 and 50.0% at day 14, and then tended to stabilize for the remainder of the 20-day experimental period. The present results indicate that combining a CO_2_ laser with albumin-shelled MBs can increase skin permeability so as to enhance the delivery of β-arbutin to inhibit melanogenesis in mice without damaging the skin.

## 1. Introduction

Cavitation refers to the formation of cavities in a liquid, and usually occurs when the liquid is subjected to rapid pressure changes. Such pressure changes can be induced using many different methods, with acoustic cavitation being initiated when the amplitude of an applied acoustic pressure exceeds a certain threshold [1]. Acoustic cavitation involves the formation, growth, pulsation, and collapse of microbubbles (MBs) in liquids during sonication by high-intensity ultrasound (US) waves. These phenomena are believed to be responsible for the mixing, fragmentation, erosion, wetting, sonocapillary, and other effects that have various practical industrial applications [1].

US-induced cavitation of MB contrast agents also plays an important role in both diagnostic and therapeutic medical applications. US contrast agents are stabilized and coated MBs that are injected intravascularly to enhance the resolution of diagnostic US imaging [2]. There is evidence from numerous studies that the presence of MB contrast agents in the blood can decrease the threshold for various US-induced biological effects both in vitro and in vivo, such as hemolysis, capillary rupture, and sonoporation [3]. Some studies have indicated that the presence of MB contrast agents in the blood significantly reduces the threshold for US-induced premature cardiac contractions [4,5]. The resonance of MBs (stable cavitation) results in nonlinear harmonic emissions that can be utilized in MB-specific contrast imaging. The inertial cavitation and destruction of MBs can induce strong mechanical stresses that increase the permeabilities of cell membranes and the blood-brain barrier for improving the delivery of therapeutic agents. In our previous studies, we have applied inertial cavitation of MBs induced by US to enhance transdermal drug delivery (TDD), since inertial cavitation was found to result in far greater permeability enhancement of the stratum corneum compared to stable cavitation [6,7,8].

Laser irradiation is an alternative approach for enhancing drug permeation and hence facilitating drug delivery into or across the skin. When a laser pulse with an intensity above a certain threshold is focused on a liquid, explosive vaporization of the liquid also can induce MB cavitation [9,10]. The aggressive nature of laser-induced cavitation has resulted in it being utilized in a broad range of applications including cell lysis, cell membrane poration, and ocular surgery [11]. Strategies for safely improving the appearance of postoperative, atrophic, and acne scars have recently been demonstrated using fractional ablative and nonablative lasers [12]. Ablative laser skin resurfacing provides the greatest clinical improvements, but the postoperative recovery takes several weeks [13]. Nonablative laser procedures might be more suitable for patients unable or unwilling to tolerate extended postoperative healing. However, prior herpes simplex infection can reactivate after nonablative laser skin remodeling due to the intense heat produced by the laser or other light source [13].

Some methods have been developed to reduce the heat produced by laser irradiation, such as the use of contact cooling handpieces or dynamic cryogenic devices capable of delivering spurts of cooling spray of variable durations [14]. However, there is still no general consensus about which method of cooling is most effective during treatment. Moreover, unlike US, the mechanism underlying the effects of laser-induced cavitation with stabilized coated MBs in liquids remains unclear.

Laser-mediated cavitation of MB contrast agent might also be highly useful for TDD. The present study investigated the efficacy of a new laser-mediated MB cavitation method in terms of avoiding the intense heat produced by the laser and enhancing TDD both in vitro and in vivo.

## 2. Materials and Methods

### 2.1. Production of Albumin-Shelled MBs

Albumin-shelled MBs were prepared according to the procedure used in our previous studies [7,15]. In brief, albumin-shelled MBs were generated by sonication in 10 mL of a solution containing 140 mg of albumin (Octapharma, Vienna, Austria) and perfluorocarbon gas in physiological saline (pH 7.4, 0.9% sodium chloride) using a sonicator (Branson Ultrasonics, Danbury, CT, USA) for 2 min. The number of perfluorocarbon-filled albumin MBs in the solution was measured using the MultiSizer III device (Beckman Coulter, Fullerton, CA, USA) with a 30-µm-aperture probe and measurement boundaries of 0.6–20 µm. The size distribution in the suspension was measured based on dynamic light scattering (Zetasizer Nano, ZS90, Malvern, UK), which revealed that the albumin-shelled MBs had a diameter of 1.02 ± 0.11 μm (mean ± SD) and a concentration of 1.40 × 10^8^ MBs/mL.

### 2.2. Laser-Induced MB Disruption

Previous studies have suggested that US-mediated MB disruption (i.e., inertial cavitation) is required for effective TDD [8,16,17]. The present study measured the efficacy of MB disruption when using different types of lasers under different conditions. The concentration of MBs was adjusted to 2.8 × 10^7^ MBs/mL (fivefold dilution) and 1.4 × 10^7^ MBs/mL (tenfold dilution) in an Eppendorf tube, and irradiation was provided by four types of laser: Air-cooled argon-ion laser (515 nm, continuous wave), supercontinuum fiber laser (1064 nm, pulsed wave), Nd:YAG laser (532 nm, pulsed wave), and CO_2_ fractional laser (10,600 nm, pulsed wave). The detailed conditions for the various types of lasers are listed in Table 1.

**Figure 1:**
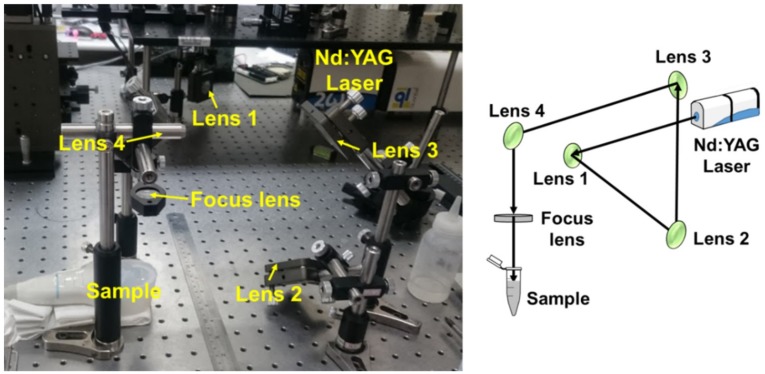
A Nd:YAG laser setup for measuring laser-induced microbubble (MB) disruption.

An example of the optical setup is shown in Figure 1. The change in temperature during laser irradiation was measured using a thermometer (Optris LS, Optris, Berlin, Germany). Then, after exposure to the laser, 100 μL of the MB solution was added to a microscope slide for observation. The numbers of MBs in light-microscopy images before and after laser irradiation were converted into 8-bit grayscale images using MATLAB (The MathWorks, Natick, MA, USA) to facilitate the observations of MB disruption. The destruction rate of MBs was quantified according to the damaged areas using the following equation:

(1) $$R_{d}\;(\%)=\frac{A_{1}-A_{i}}{A_{1}}\times 100\%$$

where *A*_1_ and *A_i_* are the numbers of black pixels (corresponding to the amounts of MBs) immediately before and after the laser irradiation, respectively.

### 2.3. Penetration Depth in Pigskin

The enhancement of penetration depth achievable using laser-mediated MB cavitation was measured using pigskin. Fresh pigskin was obtained from the slaughterhouse affiliated to New Taipei City Meat Market, and all experiments on the skin samples were completed within 6 h. Circular porcine ear skin samples with a radius of 1.5 cm and a thickness of 3 mm were produced. They were encircled with gel to prevent leakage and then loaded with Evans blue or MBs. Before laser irradiation, 500 μL of MBs was loaded into the treatment area of the sample and then irradiated by an Nd:YAG laser and CO2 fractional laser attached to the top of the sample. Each type of laser was applied successively for different concentrations of MBs. After removing the saline solution or MBs, 500 μL of Evans blue (0.10% w/w) was added and left for 15 min, and then the area was washed with PBS three times for 1 min each.

The treated areas of pigskin were then embedded in an optimal-cutting-temperature solution (Surgipath FSC 22, Leica Microsystems, Buffalo Grove, IL, USA) on round specimen disks with a diameter of 2.2 cm. The embedded samples were placed on the −25 °C freezing stage of a cryostat (Microm HM550 series, Thermo Fisher Scientific, Bremen, Germany) for about 30 min, and transverse sectioning was then performed at a slice thickness of 10 μm. Sections attached to the microscopy slides were air-dried at room temperature and mounted for microscopy examinations. The distribution of the Evans blue in the cryosections was determined with the aid of an upright microscope (DM 2500, Leica Microsystems). The results obtained for the penetration depth in pigskin indicated that the optimal MB concentration was used for the CO_2_ fractional laser, and so this was used in the subsequent in vitro and in vivo experiments.

### 2.4. In Vitro Skin Penetration by β-arbutin Solution

A 2-mm-thick sample of pigskin was harvested using a Humby knife, carefully cleaned with PBS, and cut into square pieces (2 cm × 2 cm). Circular areas on the skin samples with a radius of 1.5 cm and a height of 5 mm were encircled with gel to prevent leakage when the sample was loaded with 500 μL of MBs. After irradiating the sample seven times (the conditions are listed in Table 1) with a CO_2_ fractional laser, skin penetration was tested using static Franz diffusion cells over an area of 2.14 cm^2^ according to the experimental design that we have described previously [7]. The temperature of the diffusion assembly was maintained at 37 °C. β-arbutin (30 mg/mL, 500 μL, 4-hydroxyphenyl-β-d-glucopyranoside, molecular mass = 272.25 Da; Sigma-Aldrich, St. Louis, MO, USA) was applied to the epidermal side of the skin and occluded with parafilm (Pechiney Laboratory Safety Products and Apparel, Chicago, IL, USA). The receptor diffusion compartment facing the dermal side was filled with 12 mL of PBS (pH 7.4), which was stirred by a magnetic bar rotating at 600 rpm. Test solutions that did not contain MBs were filtered through a 0.2-μm micropore filter (Nalgene, Rochester, NY, USA) or a 0.22-μm micropore filter (Millex, Darmstadt, Germany). Aliquots (200 μL) of the receptor solution were taken at 0, 1, 2, 3, 4, 6, 8, 10, 12, and 24 h, and replaced by the same volume of fresh receptor solution.

The obtained samples were kept in a freezer until analyzed by high-performance liquid chromatography (HPLC). At the end of the penetration experiments (after 24 h), the skin sample was detached from the diffusion cells, carefully rinsed five times with distilled water to remove excess β-arbutin from its surface, cut into 0.1-g pieces, and homogenized with 1 mL of receptor solution for 2 min at 10,000 rpm (Polytron-Aggregate PT3100, Kinematica, Luzern, Switzerland). The homogenized suspension was centrifuged twice for 25 min at 3100× *g* (Thermo Fisher Scientific) and 4 °C prior to determining the concentrations of β-arbutin in the supernatant using HPLC.

### 2.5. HPLC Analysis of β-arbutin

An Inspire™ C18 column (250 mm × 4.6 mm, 5 μm particle size; Dikma Technologies, Lake Forest, CA, USA) was used to measure the β-arbutin concentrations. The HPLC system was equipped with a binary pump (PU-2089, Jasco, Tokyo, Japan), and the wavelength of the ultraviolet (UV) detector (UV-2075, Jasco) was set at 280 nm. The mobile phase consisted of methanol:distilled water (pH 5.5, 70:30 *v*/*v*) [18] at a flow rate of 0.6 mL/min. All samples to be analyzed were injected at a volume of 20 μL. The retention time of β-arbutin was about 4.3 min.

### 2.6. Animal Treatments

The melanin content of origanoside was investigated in the C57BL/6J mouse model [19]. Five-week-old mice weighing 20–25 g were obtained from Bio Lasco (Taipei, Taiwan). The experimental protocol was approved by the Institutional Animal Care and Use Committee of the National Defense Medical Center, Taipei, Taiwan. The procedures for animal care complied with institutional guidelines and regulations (approval no. IACUC-17-092). Throughout the experiments, the animals were housed in stainless-steel cages in an air-conditioned room with the temperature maintained at 25–28 °C and with alternating light and dark cycles of 12 h each.

The animals were acclimatized for 7 days prior to the experiment. After their hair had been removed from an area of 2 cm × 2 cm, the skin color was measured using a chroma meter (CR-400, Konica Minolta Sensing, Tokyo, Japan). The animals were then exposed to ultraviolet B (UVB) radiation (G8T5E, Sankyo, Tokyo, Japan) to induce hyperpigmentation (total energy dose per exposure = 1 J/cm^2^, wavelength = 306 nm, three times per week for 2 weeks), and then the skin color was measured again. 

The animals were divided into the following five groups (*n* = 5 per group, treatment applied once every 3 days for 20 days): (1) no treatment (group C); (2) application of penetrating β-arbutin alone (300 μg/mL, 0.2 mL/cm^2^) (group A); (3) laser irradiating the skin directly with the application of penetrating β-arbutin (300 μg/mL, 0.2 mL/cm^2^) (group L + A); (4) laser irradiating the skin covered by saline and with the application of penetrating β-arbutin (300 μg/mL, 0.2 mL/cm^2^) (group L + S + A); and (5) laser irradiating the skin combined with MBs on the skin and with the application of penetrating β-arbutin (300 μg/mL, 0.2 mL/cm^2^) (group L + MBs + A). The change in skin color induced by each of the treatments was assessed at predetermined times using the chroma meter. The luminosity index, *L* [20], was calculated on each measurement day before and after treatment.

### 2.7. Histological Study

Skin tissue samples (approximately 8 mm × 8 mm) were taken from the treatment area immediately after the experiments and stored in a 10% formalin solution. Hematoxylin and eosin (HE; Sigma-Aldrich) staining was applied, and the samples were analyzed by an expert dermatopathologist (H.W.G.). Some other samples were stained with Fontana-Masson silver nitrate (Kojima Chemical, Kashiwabara, Japan) for 30 min at 60 °C, and then washed with distilled water and fixed in 5% sodium thiosulfate solution (Duksan, Seoul, Korea) for 2 min, before washing again with distilled water. The samples were then stained with nuclear fast red solution (Fluka, Buchs, Switzerland) for 5 min and washed twice with distilled water. Finally, the samples were dehydrated in 95% followed by 100% ethanol, and then washed twice with xylene (Duksan) [21].

### 2.8. Statistical Analysis

The obtained data were analyzed statistically using Student’s *t*-test. A probability value of *p* < 0.05 was considered indicative of a significant difference.

## 3. Results

### 3.1. Laser-Induced MB Disruption

**Figure 2:**
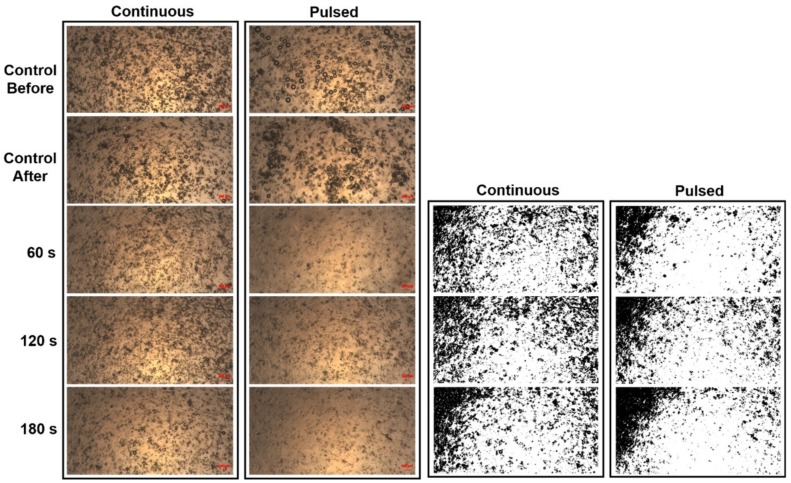
Microscopy images (two leftmost columns) of fivefold-diluted MBs without and with continuous- and pulsed-laser irradiation at 60, 120, and 180 s. The controls labeled as before and after refer to the original (fivefold-diluted) MBs and the MBs remaining on the slide after 180 s in the absence of laser irradiation. The microscopy images in the two rightmost columns are the images of the corresponding leftmost columns converted into grayscale images.

**Figure 3:**
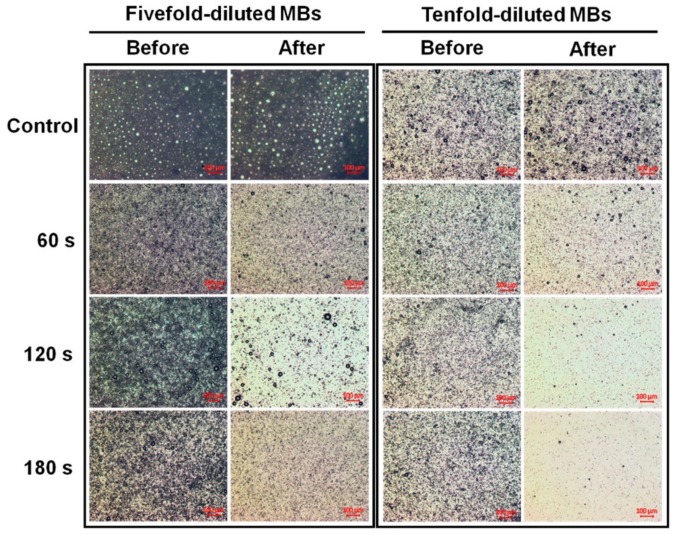
Microscopy images of five- and tenfold-diluted MBs without and with Nd:YAG pulsed-laser irradiation at 60, 120, and 180 s.

**Figure 4:**
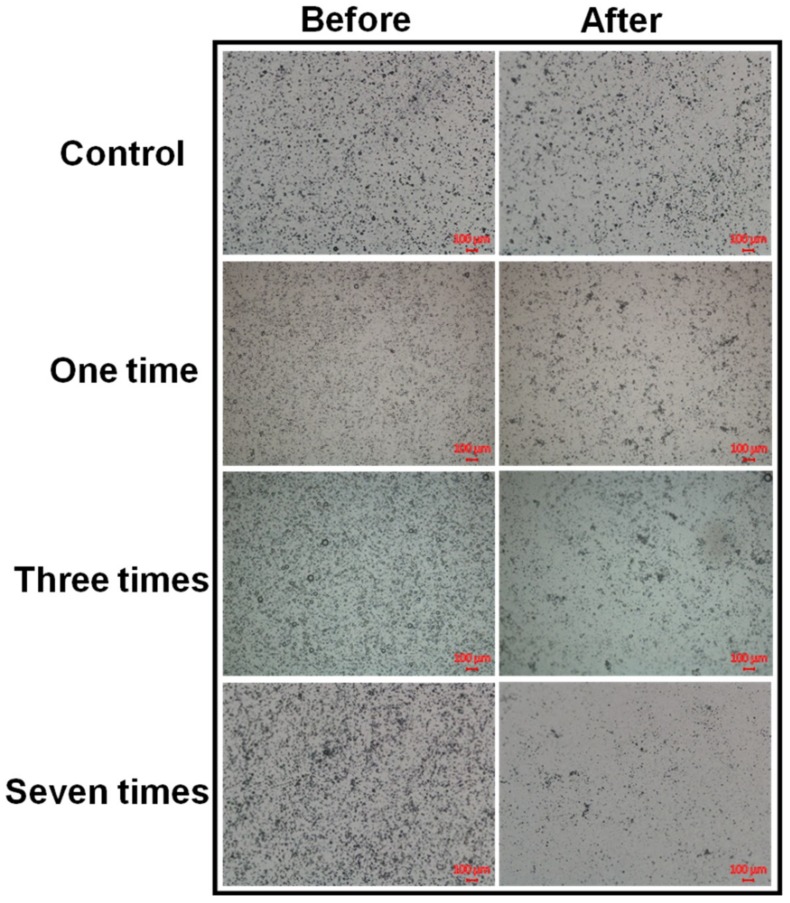
Microscopy images of tenfold-diluted MBs without and with irradiation by a clinical CO_2_ fractional pulsed laser one, three, and seven times.

Microscopy images of fivefold-diluted MBs without and with irradiation by a 10.8 mW continuous (air-cooled argon-ion) laser and a 10.8 mW pulsed (supercontinuum fiber) laser for 60, 120, and 180 s are shown in Figure 2. The destruction rates for the pulsed laser increased by 17.66%, 20.52%, and 39.05% compared to the continuous laser at 60, 120, and 180 s, respectively. Figure 3 shows the destruction efficacy of five- and tenfold-diluted MBs when using an Nd:YAG pulsed laser at 60, 120, and 180 s. The destruction rates of five- and tenfold-diluted MBs were 72.46% and 78.59%, respectively, at 60 s, 88.06% and 96.10% at 120 s, and 85.22% and 98.80% at 180 s. Figure 4 shows the destruction efficacy of tenfold-diluted MBs for being irradiated one, three, and seven times by the clinical CO_2_ fractional pulsed laser. The destruction rate increased with the irradiation time, being close to 100% for seven-times irradiation, and so this condition was used in the subsequent in vitro and in vivo experiments.

### 3.2. Penetration Depth in Pigskin

**Figure 5:**
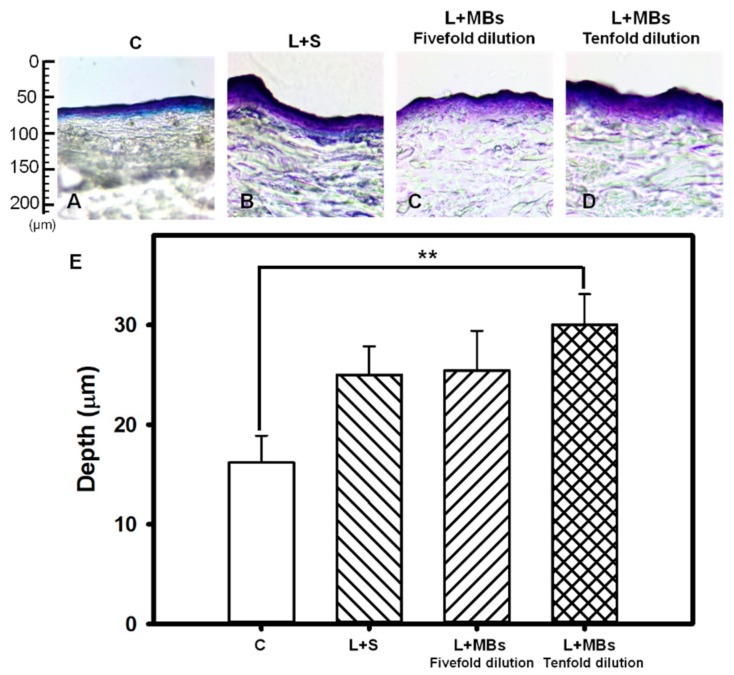
Light-microscope evaluation of pigskin samples with no treatment (group C) (**A**); for irradiation by the Nd:YAG pulsed laser combined with saline (group L + S) (**B**); and for laser irradiation combined with MBs (group L + MBs) diluted fivefold (**C**) and tenfold (**D**); (**E**) Quantification of the penetration depths of Evans blue in panels (**A**–**D**) (** *p* < 0.01). Data are mean and SD values.

The pigskin samples with no treatment (group C) and those covered with saline, fivefold-diluted MBs, and tenfold-diluted MBs after irradiating with the Nd:YAG pulsed laser are shown in Figure 5. Figure 5E quantifies the penetration depths in the four groups (*n* = 4). The degree of penetration in both the cuticle and the dermis was significantly greater for tenfold-diluted MBs than for the other groups, and did not differ significantly between laser treatments applied to the samples covered with saline and fivefold-diluted MBs. The overall penetration depth in group control was 16.19 ± 2.71 μm, and this increased to 25.0 ± 2.87, 25.4 ± 3.97, and 30.03 ± 3.07 μm in the saline, fivefold-diluted MBs, and tenfold-diluted MBs groups, respectively, irradiated by the laser. The penetration depth and uniformity were both greatest for tenfold-diluted MBs, and so this condition was used in the subsequent experiments involving the in vitro penetration depth in pigskin and the in vivo animal treatments.

**Figure 6:**
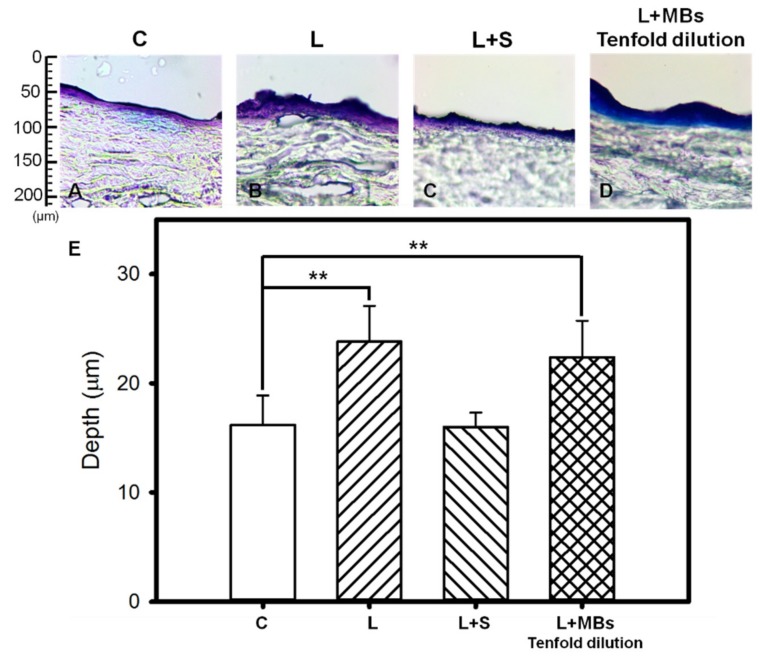
Light-microscope evaluation of pigskin samples in group C (**A**); for irradiation by the CO_2_ fractional pulsed laser directly (group L) (**B**); and for laser irradiation combined with saline (group L + S) (**C**) and tenfold-diluted MBs (group L + MBs) (**D**); (**E**) Quantification of the penetration depths of Evans blue in panels (**A**–**D**) (** *p* < 0.01). Data are mean and SD values.

**Figure 7:**
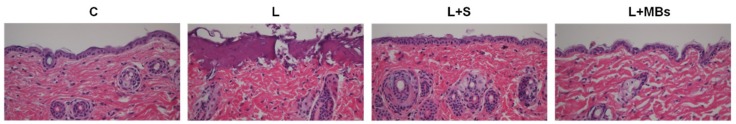
Light-microscope hematoxylin and eosin (HE) evaluation of pigskin samples in group C, for irradiation with the CO_2_ fractional pulsed laser directly (group L), and for laser irradiation combined with saline (group L + S) and tenfold-diluted MBs (group L + MBs).

Figure 6 shows that when using the clinical CO_2_ fractional pulsed laser, the degree of penetration in both the cuticle and the dermis was significantly greater for tenfold-diluted MBs group (22.38 ± 3.35 μm) and laser direct irradiation (23.82 ± 3.26 μm) than for the other groups, and did not differ significantly between the saline group with laser irradiation (16.00 ± 1.33 μm) and the control group (16.19 ± 2.71 μm). However, Figure 7 shows that damage to both the cuticle and the dermis was more obvious for direct laser irradiation in HE-stained microscopy images.

### 3.3. In Vitro Skin Penetration by the β-arbutin Solution

**Figure 8:**
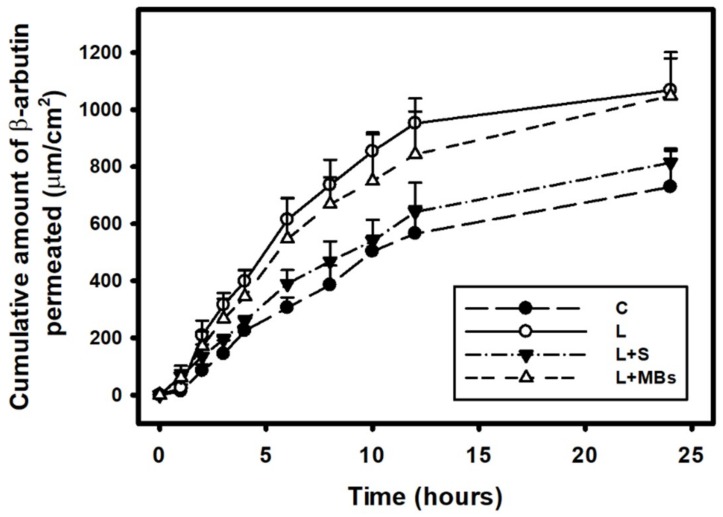
In vitro drug penetration in the different experimental groups (see Table 2) through pigskin in a Franz diffusion cell at 36–37 °C. Data are mean and SD values.

Figure 8 shows the β-arbutin concentrations in the four groups for percutaneous penetration over 24 h as analyzed using HPLC. The concentration in all groups increased rapidly during the first 12 h and then gradually leveled off from 12 to 24 h. At 24 h, the concentration was significant higher (*p <* 0.05) for laser irradiation only (group L) (1067.97 ± 111.68 μg/mL) and for laser irradiation combined with MBs (group L + MBs) (1048.03 ± 153.35 μg/mL) than for laser irradiation combined with saline (group L + S) (814.61 ± 41.29 μg/mL) and β-arbutin alone (group C) (729.45 ± 133.57 μg/mL). The concentration did not differ significantly (*p* < 0.05) between groups L and L + MBs, or between groups L + S and C. The penetration and deposition of β-arbutin at 6 h were 2.0 and 1.8 times higher in groups L + MBs and L, respectively, than in group C. Table 2 indicates that the amount of β-arbutin deposited in the skin was higher in groups L + S and L + MBs than in groups C and L at 24 h (*p* < 0.01). The total amount of β-arbutin that penetrated was significantly greater in group L + MBs than in the other three groups.

### 3.4. Animal Treatments

**Figure 9:**
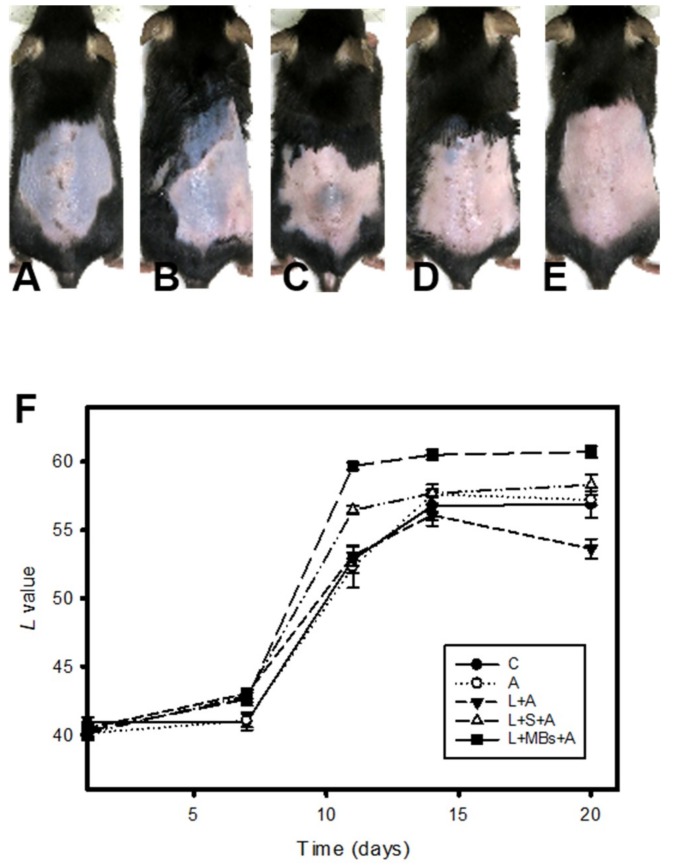
Photographs of mouse skin in group C (**A**); for the application of penetrating β-arbutin alone (group A) (**B**); for the laser irradiating the skin directly with the application of penetrating β-arbutin (group L + A) (**C**); for the laser irradiating the skin covered by saline and with the application of penetrating β-arbutin (group L + S + A) (**D**); and for the laser irradiating the skin combined with MBs on the skin and with the application of penetrating β-arbutin (group L + MBs + A) (**E**) over a 20-day treatment period; (**F**) Quantification of the skin-whitening effects of β-arbutin on ultraviolet-radiation-induced hyperpigmentation for the groups in panels (**A**–**E**). Data are mean and SD values.

Figure 9 shows photographs of mouse skin after UVB exposure in a completely untreated animal (Figure 9A) and in groups A (Figure 9B), L + A (Figure 9C), L + S + A (Figure 9D), and L + MBs + A (Figure 9E) at day 20. The skin brightness was more effectively increased and closer to the original color in group L + MBs + A than in groups A, L + A, and L + S + A. Figure 9F plots the brightness (i.e., *L*) values to demonstrate the whitening effects of β-arbutin on UV-induced hyperpigmentation over 20 days. The brightness value (which had a possible range of 0–100) was around 40 in each group after UVB exposure. At day 11 the brightness value in group L + MBs + A had increased by 48.1%. There were significant skin-whitening effects (*p* < 0.05) in groups L + S + A and L + MBs + A compared to the other groups, but not in groups C, A, and L + A (Bonferroni *p* > 0.05). At day 11 the brightness values in groups C, A, L + A, L + S + A, and L + MBs + A had increased by 27.6%, 30.4%, 32.1%, 40.6%, and 48.1%, respectively. At day 14 the increase in the brightness value in group L + MBs + A had reached a plateau of 50.1%, making it close to the original skin color, while the increases in groups C, A, L + A, and L + S + A plateaued at the smaller values of 38.9%, 43.6%, 39.3%, and 43.9%, respectively. The brightness value before UVB exposure was 60.76 ± 0.41, and after 20 days it was only close to this value in group L + MBs + A.

**Figure 10:**
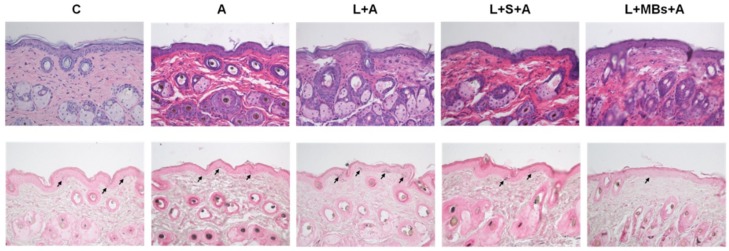
Histology images (HE, (**upper row**); Fontana-Masson silver nitrate, (**lower row**)) for groups C, A, L + A, L + S + A, and L + MBs + A at day 20. Arrows indicate melanocytes in the basal layer of the epidermis.

The results of the histopathology analysis in Figure 10 reveal that there was a significant decrease in the relative melanin content in group L + MBs + A. No damage to skin structures or bilayer-bilayer interfaces was observed in any of the treatment groups.

## 4. Discussion

The inertial cavitation of MBs induced by US produces much greater permeability enhancement of the stratum corneum compared to stable cavitation. This study measured laser-induced MB disruption under various conditions with the aim of identifying the ideal condition for generating inertial cavitation. Some previous studies have found that interactions between a pulsed laser and liquid results in the formation of MB cavitation [22]. It was realized that short and ultrashort pulsed laser induced cavitation offer simpler and better controlled conditions of bubble cavitation due to the optical breakdown [23]. Continuous lasers induced cavitation has been reported causing by thermal expansion and liquid boiling [24]. Figure 2 shows that the distribution of MBs in microscopy images was more inhomogeneous for the pulsed laser than for the continuous laser. Moreover, at the same laser output power, there were significantly fewer MBs for the pulsed laser than for the continuous laser. This indicates that when a liquid already contains stable MBs, without raising the temperature, irradiation by a pulsed laser induces more stress waves that can disrupt more MBs for inducing inertial cavitation compared to when using a continuous laser.

Figure 3 and Figure 4 show that significant disruption occurred for the tenfold-diluted MBs after either 180 s of pulsed-laser irradiation or seven applications of CO_2_ fractional pulsed-laser irradiation and without any significant increase in temperature, indicating that inertial cavitation was produced effectively under these conditions. Consistently, Figure 5 and Figure 6 indicated that the penetration depth of Evans blue was greater for the tenfold-diluted MBs groups than for the other groups, and was proportional to the degree of MB rupture. These results indicate that the laser-induced inertial cavitation of MBs could also play an important role in TDD. Figure 6 and Figure 7 show that although the penetration depth of Evans blue in group L was similar to that in group L + MBs, some damage did occur in the stratum corneum. MBs might therefore also act as a buffer for reducing damage during laser irradiation.

CO_2_ and Er:YAG lasers reportedly facilitate drug delivery, and the CO_2_ laser is one of the most widely used lasers in the dermatology field for ablating benign raised lesions. Although the longer wavelength of CO_2_ laser radiation results in deeper penetration, it also generates more heat [25,26]. Moreover, the high-water content of soft tissue both makes it an excellent target for the CO_2_ laser operating at 10,600 nm and also offers a degree of inherent safety because of its high-water absorption [27]. Figure 8 and Table 1 indicate that although the temperature increase was only 1.1 °C with the saline and MB solutions absorbed the incident CO_2_ laser irradiation, the total amount of β-arbutin that penetrated the skin was greater in group L + MBs than in group L + S. This indicates that the efficacy of laser-induced TDD is greater when the liquid already contains stable MBs. It is also consistent with the results found in the C57BL/6J mouse model. At day 11, the brightness values in groups L + MBs + A and L + S + A had increased more significantly (by 48.1% and 40.6%, respectively) than in the other three groups. The brightness value was still more obvious in group L + MBs + A than in group L + S + A. These results indicate that more laser-induced cavitation occurs in a liquid containing stabilized MBs than in a liquid alone. The laser-mediated cavitation of MB contrast agent can enhance TDD while avoiding the production of intense heat. Moreover, the duration when irradiating seven times with a CO_2_ fractional pulsed laser was shorter than when using US (1 min, according to our previous studies) [6,7]. Based on the dynamic cryogenic devices that deliver spurts of cooling spray of variable durations which have been developed to reduce the heating effect during laser irradiation [14], sprays that contain stabilized MBs could induce inertial cavitation to enhance TDD.

## 5. Conclusions

This study has produced a novel laser-mediated TDD platform for facilitating drug delivery based on utilizing laser-mediated MB cavitation. When a liquid already contains stable coated MBs, irradiation by a pulsed laser induces stress waves that can disrupt more MBs for inducing inertial cavitation compared to when using a continuous laser. Moreover, the inertial cavitation of MBs induced by a pulsed laser could play an important role in TDD. The results obtained in the present in vitro and in vivo experiments indicated that laser-induced cavitation with stabilized MBs in a liquid could enhance TDD more than when using a liquid alone. Moreover, this enhancement of TDD occurs without the production of intense heat, and so the MBs might also act as a buffer for reducing damage during laser irradiation.

## Figures and Tables

**Table 1:** Conditions and parameters of the argon-ion laser, supercontinuum fiber laser, Nd:YAG laser, and CO_2_ fractional laser used when measuring laser-induced microbubble (MB) disruption.

Laser Type	Argon Ion Laser	Supercontinuum Fiber	Nd:YAG	CO_2_ Fractional
Wavelength (nm)	515	1064	532	10,600
Mode of operation	Continuous	Pulsed	Pulsed	Pulsed
Energy (mJ)	n/a	n/a	380	1.3
Output power (mW)	10.8	10.8	n/a	n/a
Repetition rate	n/a	80 MHz	5 Hz	Single
Radiation repetitions (in vivo)	n/a	n/a	n/a	Seven times
Radiation times/repetitions (in vitro)	60, 120, or 180 s	60, 120, or 180 s	60, 120, or 180 s	One, three, or seven times
MB dilution	Fivefold	Fivefold	Five- or tenfold	Tenfold
Average temperature increase (°C)	0.7	1.0	0.86 or 0.23	1.1

**Table 2:** Permeated amount of β-arbutin after 24 h, deposited on the skin, and penetrated across the skin. Data are mean ± SD values. C, penetrating β-arbutin alone; L, laser combined with penetrating β-arbutin; L + S, laser combined with saline and penetrating β-arbutin; L + MBs, laser combined with MBs and penetrating β-arbutin.

Group	Skin Weight (mg)	Amount of β-arbutin Penetrated Across Skin (mg/mL)	Amount of β-arbutin Deposited on Skin (mg/mL)	Total Amount of β-arbutin Penetrated (mg/mL)
C	18.88 ± 3.30	2.397 ± 0.07	2.291 ± 0.419	4.688 ± 0.491
L	23.43 ± 0.93	4.876 ± 0.017	2.559 ± 0.129	7.435 ± 0.146
L + S	18.75 ± 3.54	3.287 ± 0.01	3.355 ± 0.350	6.642 ± 0.451
L + MBs	22.47 ± 1.10	4.892 ± 1.14	3.292 ± 0.481	8.184 ± 1.628

## References

[1] Tzanakis I., Lebon G.S., Eskin D.G., Pericleous K.A. (2017). Characterizing the cavitation development and acoustic spectrum in various liquids. Ultrason. Sonochem..

[2] Dalecki D., Emilio Q. (2005). Biological Effects of Microbubble-Based Ultrasound Contrast Agents. Contrast Media in Ultrasonography: Basic Principles and Clinical Applications.

[3] Rota C., Raeman C.H., Child S.Z., Dalecki D. (2006). Detection of acoustic cavitation in the heart with microbubble contrast agents in vivo: A mechanism for ultrasound-induced arrhythmias. J. Acoust. Soc. Am..

[4] Van der Wouw P.A., Brauns A.C., Bailey S.E., Powers J.E., Wilde A.A. (2000). Premature ventricular contractions during triggered imaging with ultrasound contrast. J. Am. Soc. Echocardiogr..

[5] Li P., Cao L.Q., Dou C.Y., Armstrong W.F., Miller D. (2003). Impact of myocardial contrast echocardiography on vascular permeability: An in vivo dose response study of delivery mode, pressure amplitude and contrast dose. Ultrasound Med. Biol..

[6] Liao A.H., Lu Y.J., Hung C.R., Yang M.Y. (2016). Efficacy of transdermal magnesium ascorbyl phosphate delivery after ultrasound treatment with microbubbles in gel-type surrounding medium in mice. Mater. Sci. Eng. C Mater. Biol. Appl..

[7] Liao A.H., Ma W.C., Wang C.H., Yeh M.K. (2016). Penetration depth, concentration and efficiency of transdermal α-arbutin delivery after ultrasound treatment with albumin-shelled microbubbles in mice. Drug Deliv..

[8] Oberli M.A., Schoellhammer C.M., Langer R., Blankschtein D. (2014). Ultrasound-enhanced transdermal delivery: Recent advances and future challenges. Ther. Deliv..

[9] Paltauf G., Schmidt-Kloiber H. (1996). Microcavity dynamics during laser-induced spallation of liquids and gels. Appl. Phys. A.

[10] Vogel A., Noack J., Nahen K., Theisen D., Busch S., Parlitz U., Hammer D.X., Noojin G.D., Rockwell B.A., Birngruber R. (1999). Energy balance or optical breakdown in water at nanosecond to femtosecond time scales. Appl. Phys. B.

[11] Goldberg D.J., Cutler K.B. (2000). Nonablative treatment of rhytides with intense pulsed light. Lasers Surg. Med..

[12] Jang J.U., Kim S.Y., Yoon E.S., Kim W.K., Park S.H., Lee B.I., Kim D.W. (2016). Comparison of the effectiveness of ablative and non-ablative fractional laser treatments for early stage thyroidectomy scars. Arch. Plast. Surg..

[13] Metelitsa A.I., Alster T.S. (2010). Fractionated laser skin resurfacing treatment complications: A review. Dermatol. Surg..

[14] Kelly K.M., Nelson J.S., Lask G.P., Geronemus R.G., Bernstein L.J. (1999). Cryogen spray cooling in combination with nonablative laser treatment of facial rhytides. Arch. Dermatol..

[15] Liao A.H., Lu Y.J., Lin Y.C., Chen H.K., Sytwu H.K., Wang C.H. (2016). Effectiveness of a layer-by-layer microbubbles-based delivery system for applying minoxidil to enhance hair growth. Theranostics.

[16] Prausnitz M.R., Langer R. (2008). Transdermal drug delivery. Nat. Biotechnol..

[17] Liao A.H., Hung C.R., Chen H.K., Chiang C.P. (2018). Ultrasound-mediated EGF-coated-microbubble cavitation in dressings for wound-healing applications. Sci. Rep..

[18] Wen A.H., Choi M.K., Kim D.D. (2006). Formulation of liposome for topical delivery of arbutin. Arch. Pharm. Res..

[19] Ishikawa M., Kawase I., Ishii F. (2006). Glycine inhibits melanogenesis in vitro and causes hypopigmentation in vivo. Biol. Pharm. Bull..

[20] Tsai Y.H., Lee K.F., Huang Y.B., Huang C.T., Wu P.C. (2010). In vitro permeation and in vivo whitening effect of topical hesperetin microemulsion delivery system. Int. J. Pharm..

[21] Chung S.Y., Seo Y.K., Park J.M., Seo M.J., Park J.K., Kim J.W., Park C.S. (2009). Fermented rice bran downregulates MITF expression and leads to inhibition of α-MSH-induced melanogenesis in B16F1 melanoma. Biosci. Biotechnol. Biochem..

[22] Quinto-Su P.A., Venugopalan V., Ohl C.D. (2008). Generation of laser-induced cavitation bubbles with a digital hologram. Opt. Express.

[23] Ramirez-San-Juana J.C., Rodriguez-Aboytesa E., Korneeva N., Baldovinos-Pantaleona O., Chiu-Zarateb R., Gutiérrez-Juárezb G., Dominguez-Cruzc R., Ramos-Garciaa R. Cavitation Induced by Continuous Wave Lasers. Proceedings of the SPIE Optical Trapping and Optical Micromanipulation IV.

[24] Rastopov S.F., Sukhodolsky A.T. Sound Generation by Thermocavitation Induced CW—Laser in Solutions. Proceedings of the SPIE Optical Radiation Interaction with Matter.

[25] Omi T., Numano K. (2014). The role of the CO2 laser and fractional CO2 laser in dermatology. Laser Ther..

[26] Zaleski-Larsen L.A., Fabi S.G. (2016). Laser-assisted drug delivery. Dermatol. Surg..

[27] Lin C.H., Aljuffali I.A., Fang J.Y. (2014). Lasers as an approach for promoting drug delivery via skin. Expert. Opin. Drug Deliv..

